# Differences in Brain Volume in Military Service Members and Veterans After Blast-Related Mild TBI: A LIMBIC-CENC Study

**DOI:** 10.1001/jamanetworkopen.2024.43416

**Published:** 2024-11-11

**Authors:** Emily L. Dennis, Jared A. Rowland, Carrie Esopenko, Nicholas J. Tustison, Mary R. Newsome, Elizabeth S. Hovenden, Brian B. Avants, Jessica Gill, Sidney R. Hinds, Kimbra Kenney, Hannah M. Lindsey, Sarah L. Martindale, Mary Jo Pugh, Randall S. Scheibel, Pashtun-Poh Shahim, Robert Shih, James R. Stone, Maya Troyanskaya, William C. Walker, Kent Werner, Gerald E. York, David X. Cifu, David F. Tate, Elisabeth A. Wilde

**Affiliations:** 1 Department of Neurology, University of Utah School of Medicine, Salt Lake City; 2 George E. Wahlen Veterans Affairs Medical Center, Salt Lake City, Utah; 3 W. G. (Bill) Hefner VA Healthcare System, Salisbury, North Carolina; 4 Department of Translational Neuroscience, Wake Forest School of Medicine, Winston-Salem, North Carolina; 5 Department of Rehabilitation and Human Performance, Icahn School of Medicine at Mount Sinai, New York, New York; 6 Department of Radiology and Medical Imaging, University of Virginia, Charlottesville; 7 H. Ben Taub Department of Physical Medicine and Rehabilitation, Baylor College of Medicine, Houston, Texas; 8 National Institutes of Health, National Institute of Nursing Research, Bethesda, Maryland; 9 Center for Neuroscience and Regenerative Medicine, Uniformed Services University, Bethesda, Maryland; 10 Department of Neurology, Uniformed Services University, Bethesda, Maryland; 11 National Intrepid Center of Excellence, Walter Reed National Military Medical Center, Bethesda, Maryland; 12 Department of Medicine, University of Utah School of Medicine, Salt Lake City; 13 Information Decision-Enhancement and Analytic Sciences Center, VA Salt Lake City, Salt Lake City, Utah; 14 Michael E. DeBakey Veterans Affairs Medical Center, Houston, Texas; 15 Rehabilitation Medicine Department, National Institutes of Health Clinical Center, Bethesda, Maryland; 16 Department of Radiology and Radiological Sciences, Uniformed Services University, Bethesda, Maryland; 17 Department of Physical Medicine and Rehabilitation, Virginia Commonwealth University, Richmond; 18 Richmond Veterans Affairs Medical Center, Central Virginia VA Healthcare System, Richmond; 19 Alaska Radiology Associates, Anchorage

## Abstract

**Importance:**

Blast-related mild traumatic brain injuries (TBIs), the “signature injury” of post-9/11 conflicts, are associated with clinically relevant, long-term cognitive, psychological, and behavioral dysfunction and disability; however, the underlying neural mechanisms remain unclear.

**Objective:**

To investigate associations between a history of remote blast-related mild TBI and regional brain volume in a sample of US veterans and active duty service members.

**Design, Setting, and Participants:**

Prospective cohort study of US veterans and active duty service members from the Long-Term Impact of Military-Relevant Brain Injury Consortium–Chronic Effects of Neurotrauma Consortium (LIMBIC-CENC), which enrolled more than 1500 participants at 5 sites used in this analysis between 2014 and 2023. Participants were recruited from Veterans Affairs medical centers across the US; 774 veterans and active duty service members of the US military met eligibility criteria for this secondary analysis. Assessment dates were from January 6, 2015, to March 31, 2023; processing and analysis dates were from August 1, 2023, to January 15, 2024.

**Exposure:**

All participants had combat exposure, and 82% had 1 or more lifetime mild TBIs with variable injury mechanisms.

**Main Outcomes and Measures:**

Regional brain volume was calculated using tensor-based morphometry on 3-dimensional, T1-weighted magnetic resonance imaging scans; history of TBI, including history of blast-related mild TBI, was assessed by structured clinical interview. Cognitive performance and psychiatric symptoms were assessed with a battery of validated instruments. We hypothesized that regional volume would be smaller in the blast-related mild TBI group and that this would be associated with cognitive performance.

**Results:**

A total of 774 veterans (670 [87%] male; mean [SD] age, 40.1 [9.8] years; 260 [34%] with blast-related TBI) were included in the sample. Individuals with a history of blast-related mild TBI had smaller brain volumes than individuals without a history of blast-related mild TBI (which includes uninjured individuals and those with non–blast-related mild TBI) in several clusters, with the largest centered bilaterally in the superior corona radiata and subcortical gray and white matter (cluster peak Cohen *d* range, −0.23 to −0.38; mean [SD] Cohen *d*, 0.28 [0.03]). Additionally, causal mediation analysis revealed that these volume differences significantly mediated the association between blast-related mild TBI and performance on measures of working memory and processing speed.

**Conclusions and Relevance:**

In this cohort study of 774 veterans and active duty service members, robust volume differences associated with blast-related TBI were identified. Furthermore, these volume differences significantly mediated the association between blast-related mild TBI and cognitive function, indicating that this pattern of brain differences may have implications for daily functioning.

## Key Points

**Question:**

Are there differences in regional brain volume in military service members and veterans with a history of blast-related mild traumatic brain injury (TBI)?

**Findings:**

In this cohort study of 774 service members of the US military, individuals with a history of blast-related mild TBI had a significantly smaller volume in several central brain regions, including the corona radiata, internal capsule, and globus pallidus, compared with those who did not have a history of blast-related mild TBI. These differences in brain volume were further associated with cognitive performance.

**Meaning:**

In this study, blast-related mild TBI was associated with long-term differences in brain structure that explained the association between blast-related TBI and cognitive function, highlighting the importance of continuing care and regular assessments to track changes over time.

## Teaser

This cohort study investigates associations between a history of remote blast-related mild traumatic brain injury (TBI) and regional brain volume in a sample of US veterans and active duty service members.

## Introduction

Research on blast-related mild traumatic brain injury (TBI) has increased exponentially over the past 2 decades, advancing our understanding of mechanisms and outcomes.^1,2,3,4,5^ Modern warfare, combat training, and advances in weapons technology have exposed service members to blasts at an alarming rate, and most TBIs that occur in war zones include a blast as a mechanism of injury.^6,7^ Preclinical studies have demonstrated that primary blast forces can directly injure or impair brain structure and function in the absence of other injury mechanisms.^8,9,10^ As with all TBIs, the clinical outcomes after a blast injury are heterogeneous and only partially explained by injury-related factors. While there are obvious distinctions in the mechanism in blast vs blunt or impact TBI (hereon referred to as impact TBI), how distinct the pathophysiological alterations are, both acutely and with long-term cognitive, psychological, and functional outcomes, is less clear. A better understanding of the immediate and long-term effects of a blast-related mild TBI is crucial to improving care and developing targeted interventions for neurobehavioral sequelae.

Advanced neuroimaging has revealed structural alterations that support blasts as a unique mechanism of TBI, with white matter (WM) alterations consistently associated with blast-related mild TBI.^2,11,12,13,14,15,16,17^ Although some studies have identified group differences in specific brain regions, many report spatial heterogeneity, indicating that the pattern of disruption varies across individuals.^11,13,16,17,18^ This inconsistency is likely due to differences in the characteristics of each blast exposure, including force, direction, additional contemporaneous injury mechanisms (eg, hit with object or fall from blast energy), and protective factors (eg, protective gear and physical barriers that partially shield exposure). WM abnormalities have been associated with poor clinical outcomes in several domains, including cognition, psychiatric symptoms, and postconcussive symptoms. Additionally, these abnormalities mediate the association between mild TBI and both postconcussive symptoms and cognitive deficits.^16,17^ Veterans with blast-related mild TBI and memory disruption were found to have decreased metabolite levels in the hippocampus.^19^ Differences in either brain volume or cortical thickness have also been associated with blast exposure and blast-related mild TBI. Most studies examining cortical thickness have shown thinning associated with a history of blast exposure and blast-related mild TBI months and years after the injury,^20,21,22,23^ although 2 studies report increased cortical thickness in breachers (persons in military and law enforcement who use explosives to gain entry to buildings), with repetitive low-intensity blast exposure, albeit in small samples.^24,25^ Although fewer studies of brain volume have been published, volumetric differences have been noted in the hippocampus.^3,26^ Blast-related mild TBI has also been shown to alter brain function,^27,28^ the structure of whole-brain functional connectomes,^29^ functional connectivity throughout the brain,^30^ and functional connectivity within specific circuits.^31,32^ Further, these functional brain imaging differences have also been associated with differences in cognitive performance, specifically working memory.^33^

Together, human and animal studies suggest that blast-related mild TBI may have unique effects on the brain. However, human studies suggest significant individual heterogeneity in blast effects. Novel methods that are robust to this variability are critical for translation to clinical applications. Further, results of structural alterations following a blast-related mild TBI, particularly brain volume, are sparse and have not been associated with chronic or long-term cognitive outcomes and may suggest that current methods are not sensitive to structural differences following a blast-related mild TBI. Tensor-based morphometry (TBM) involves mapping a participant’s image to a reference image and using the resulting warp information to calculate voxelwise differences in volume. TBM offers several benefits over other approaches that measure volume, including greater reliability and power in multisite studies,^34^ and it has been applied successfully in mild TBI in prior publications but has not yet been used to study blast-related mild TBI.^35,36,37^ FreeSurfer segmentations rely on a priori regions of interest, whereas the whole-brain approach of TBM allows for a data-driven analysis. Voxel-based morphometry (VBM) requires tissue segmentation, which can be problematic in injured brains.

The objective of this study is to evaluate the association of blast-related mild TBI and brain volume on a voxelwise basis, using TBM in a large cohort of well-characterized veterans and active duty service members with remote blast-related mild TBI, impact mild TBI, or no lifetime TBI, and to identify whether alterations are associated with cognitive outcomes. We hypothesized that TBM would identify specific areas of the brain most sensitive to a blast-related mild TBI. Differences in these areas were expected to be associated with cognitive outcomes, including performance on tests of processing speed and working memory.

## Methods

### Participants

This cohort study and secondary analysis used participant data from the ongoing Long-Term Impact of Military-Relevant Brain Injury Consortium–Chronic Effects of Neurotrauma Consortium (LIMBIC-CENC) Prospective Longitudinal Study (PLS) that is described at length in prior publications.^38,39,40^ At the time of this dataset extraction, 8 sites had participated in data collection. Three sites were excluded from the current analyses due to having too few unexposed control participants for template creation (described here and in further detail in the eAppendix in Supplement 1). The institutional review boards at each site (Richmond VA Medical Center, Virginia; Michael De Bakey VA Medical Center, Houston, Texas; James A. Haley Veterans Hospital, Tampa, Florida; VA Portland Health Care System, Oregon; and Minneapolis VA Health Care System, Minnesota) approved this study. All participants provided written informed consent prior to assessment. Inclusion criteria were as follows: (1) prior military combat deployment, (2) combat exposure defined by a Deployment Risk and Resiliency Inventory Section D^41^ score higher than 1 on any item (range, 17-102, with higher scores indicating greater exposure to combat), and (3) at least 18 years of age. Exclusionary criteria were (1) moderate or severe TBI as defined by standard criteria or (2) history of a major neurologic or psychiatric disorder with a significant decrease in functional status and/or loss of ability for independent living (eg, complete spinal cord injury or schizophrenia). All participants were post-9/11 veterans and active duty service members. The Strengthening the Reporting of Observational Studies in Epidemiology (STROBE) reporting guideline was followed.

### Clinical, Neuropsychological, and Emotional Functioning Data Collection

Using a structured interview, the lifetime history of all possible concussive events was identified.^42^ Possible concussive events were assessed and classified as mild TBI vs not mild TBI according to the Department of Veterans Affairs and Department of Defense common definition.^43^ Possible concussive events were additionally characterized as occurring during deployment or outside deployment and as blast related or non–blast related, based on the mechanism of injury. Data on additional exposures to controlled and uncontrolled blasts were also collected.

#### Performance and Symptom Validity, Substance Misuse, and Emotional Functioning

Standard cutoff scores on the Medical Symptom Validity Test and Neurobehavioral Symptom Inventory Validity-10 index were used to detect suboptimal effort on neuropsychological testing and symptom overreporting, respectively.^44,45^ Alcohol consumption within the past 3 months was evaluated using the Alcohol Use Disorders Identification Test–Concise.^46^ Current posttraumatic stress disorder (PTSD) symptom severity within the past month was obtained using The PTSD Checklist for *DSM-5*,^47^ and depressive symptoms within the past 2 weeks were assessed using the Patient Health Questionnaire–9.^48^

#### Cognitive Functioning

Results from a battery of cognitive tests that have been shown to be affected after TBI in prior studies were collected.^49^ Visual memory and learning were evaluated using the Brief Visuospatial Memory Test–Revised (BVMT-R) Total Recall (range, 0-36, with higher scores indicating better recall) and Delayed Recall (range, 0-12, with higher scores indicating better recall).^50,51^ Processing speed was measured using the Wechsler Adult Intelligence Scale–Fourth Edition (WAIS-IV) Processing Speed Index (mean [SD] score, 100 [13], with higher scores indicating better performance), and mental flexibility was assessed using the Trail-Making Test (TMT)–B completion time. A derived score of the completion time of TMT-B and TMT-A was used to control for the motor speed element of the test (completion time in seconds, so 0 to infinity indicates incomplete, and more time indicates slower completion time).^52^ Working memory was measured using the WAIS-IV Digit Span subtest (range, 0-48, with higher scores indicating better performance).^53^ Raw scores were used for all cognitive measures because age and gender were included as covariates in analyses. All neuropsychological assessments were completed around the same time as the magnetic resonance imaging (MRI) scan.

### MRI Acquisition

Three-dimensional T1-weighted images were collected using a protocol recommended by the Alzheimer Disease Neuroimaging Initiative and consistent with other large TBI-based consortia.^54^ All sites implemented monitoring throughout to ensure quality and consistency. See the eTable in Supplement 1 for scan parameters.

### Tensor-Based Morphometry

A 2-step process was used for creating the study-specific template.^55^ Full methods are detailed in the eAppendix and eFigure 1 in Supplement 1. Briefly, a site-specific template was created for each of the 5 sites from 30 unexposed participants without current PTSD, semirandomly selected to be representative of the overall population. The 5 site-specific templates were merged into an overall template.

Each participant’s T1-weighted MRI scan was semiautomatically masked using antsBrainExtraction with manual corrections and intensity normalized with N4.^56^ Resulting files were registered to the site-specific templates using unbiased_pairwise_registration.^57,58^ Site-specific templates were registered to the overall template using the same algorithm. The registrations were combined to create a single warp file. The resulting warp files were converted into log jacobian determinant files, in which positive values indicate larger volumes in the participant image than the template and negative values indicate smaller volumes.

### Statistical Analysis

Assessment dates were from January 6, 2015, to March 31, 2023; processing and analysis dates were from August 1, 2023, to January 15, 2024. Voxelwise linear mixed-effects models were implemented with lme in R, version 3.1.3 (R Project for Statistical Computing) with site as a random effect and age and gender as covariates. Intracranial volume was not included as a covariate as the affine and rigid registration steps account for differences in overall brain size. Results were corrected for multiple comparisons using the searchlight false discovery rate^59^ and reported as Cohen *d*. A 2-sided *P* < .05 was considered statistically significant using the *t* test.

#### Primary and Sensitivity Analyses

Our primary group analysis compared individuals with a history of blast-related mild TBIs with those with no history of blast-related mild TBI, which included both TBI-negative individuals and those with only non–blast-related mild TBI. We reran the primary comparison with multiple potentially confounding factors included (ie, PTSD symptoms, depressive symptoms, and/or problematic alcohol use), both as categorical variables using standard cutoff scores and as continuous variables. We additionally reanalyzed covarying for years of education, number of lifetime mild TBIs, and time since injury. Within individuals with a history of deployment-related mild TBIs, we compared the individuals with blast-related mild TBI with those with impact TBI to separate the blast mechanism from the deployment context. We examined associations between several blast exposure variables and regional volume, including the number of blast-related mild TBIs and the number of blast-related possible concussive events.

#### Cognitive Analysis

We examined associations between volume and cognitive function, both across the whole sample and within the blast-related mild TBI group only. When there was cluster overlap between cognitive performance and results from the primary group comparison, we conducted a modified causal mediation analysis for each using the R package mediation.^60^ For these analyses, the initial variable was blast-related mild TBI (yes or no), the mediator was cluster volume from the primary group comparison, and the outcome variable was cognitive performance. In the modified causal approach,^61^ the total effect does not need to be significant to proceed with a mediation analysis if theoretical conditions are met, specifically that the effect of the variable on outcome is not immediate and that mediation analysis offers greater power than separate bivariate tests.

## Results

### Primary Group Analysis

**Figure 1.**
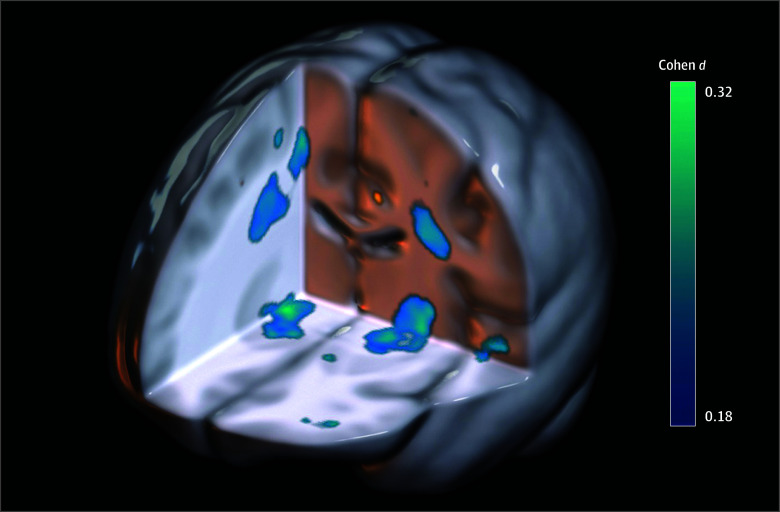
Differences in Regional Volume Between Participants With and Those Without a History of Blast-Related Mild Traumatic Brain Injury Blue clusters showing significantly smaller volumes are presented, with colors corresponding to Cohen *d*. Left in image is right in brain. Orange clusters indicate larger volumes, most of which are not visible from this view.

Table 1 reports the demographic data of the 774 participants who provided usable data (670 [86.6%] were male, and 104 [13.4%] were female; mean [SD] age, 40.1 [9.8] years). Multiple areas of smaller volume were found in the blast-related mild TBI group compared with individual with no history of blast-related mild TBI, particularly in the subcortical GM and central WM (cluster peak Cohen *d* range, −0.23 to −0.38; mean [SD] Cohen *d*, 0.28 [0.03]) (Figure 1; Table 2). For follow-up analyses, volumes were extracted for these clusters for each participant by taking the mean log jacobian determinant across the cluster mask. The bilateral superior corona radiata (SCR) clusters were merged into a single SCR mask, and the subcortical clusters, which centered on the globus pallidus and included the nucleus accumbens, substantia nigra, cerebellar peduncles, and internal capsule, were merged into a single subcortical mask.

**Table 1.** Demographic Information Across Sites

Information	5 Sites					Blast-related mild TBI		Total
	Richmond, VA (site 1)	Houston, TX (site 2)	Tampa, FL (site 3)	Portland, OR (site 6)	Minneapolis, MN (site 7)	Yes	No	
No.	252	157	205	66	94	260	514	774
Male	207	143	177	59	84	242	428	670
Female	45	14	28	7	10	18	86	104
Age, mean (SD), y	44.5 (9.6)	34.0 (7.3)	38.9 (9.2)	40 (9.5)	41.2 (10.0)	40.1 (9.8)	40.6 (10.0)	40.1 (9.8)
Type of TBI, No. (%)								
Any	192 (76.2)	125 (79.6)	162 (79.0)	56 (84.8)	63 (67.0)	NA	NA	598 (77.3)
Deployment related	115 (45.6)	102 (65.0)	112 (54.6)	29 (43.9)	33 (35.1)	NA	NA	391 (50.5)
Blast related	70 (27.8)	70 (44.6)	77 (37.6)	19 (28.8)	16 (17.0)	NA	NA	260 (33.6)
Time since last TBI, median (range), y	13.4 (0.3-51.5)	8.1 (0.4-30.4)	8.7 (0.2-50.6)	13.1 (0.6-38.1)	16.9 (0.5-48.6)	7.8 (0.2-43.6)	13.6 (0.2-51.5)	11.4 (0.2-51.5)
Education, mean (SD), y	14.5 (1.6)	13.4 (1.5)	14.1 (1.6)	14.4 (1.6)	14.6 (1.6)	14.2 (1.6)	14.3 (1.7)	14.2 (1.6)
Active duty service member, No. (%)	38 (15.1)	8 (5.1)	38 (18.5)	5 (7.6)	15 (16.0)	NA	NA	104 (13.4)
Service branch, %^a^								
Air Force	8.7	7.6	12.7	16.7	9.6	NA	NA	10.3
Army	71.8	65	67.3	54.5	73.4	NA	NA	68
Marine Corps	12.7	18.5	12.2	15.2	9.6	NA	NA	13.6
Navy	6.3	8.3	7.8	13.6	7.4	NA	NA	7.9
No answer	0.4	0.6	0	0	0	NA	NA	0.3

Abbreviations: NA, not applicable; TBI, traumatic brain injury.

^a^ Air Force, Army, Marine Corps, and Navy each include their respective Reserves and National Guard divisions.

**Table 2.** Group Differences in Brain Volume^a^

Voxels, No.	Maximum (mean) Cohen *d*	MNI coordinate			Side	Structure	Tissue
		X	Y	Z			
**Positive**							
1911	0.25 (0.21)	18	−68	−35	R	Cerebellum	WM
1425	0.32 (0.22)	30	−61	−2	R	Lingual gyrus	WM
1371	0.38 (0.25)	32	32	6	R	Inferior frontal gyrus	WM
1305	0.28 (0.22)	−34	26	1	L	Insula	GM
934	0.27 (0.21)	−24	−55	−3	L	Lingual gyrus	GM
561	0.28 (0.22)	56	−47	0	R	Middle temporal gyrus	WM
528	0.26 (0.22)	−22	−51	−48	L	Cerebellum lobule VIIIB	GM
385	0.32 (0.24)	29	15	54	R	Middle frontal gyrus	GM
336	0.27 (0.22)	4	−57	−35	R	Cerebellum vermis IX	GM
269	0.30 (0.24)	38	−8	−20	R	Middle temporal gyrus	WM
177	0.26 (0.22)	−46	−63	14	L	Middle temporal gyrus	GM
117	0.28 (0.23)	29	12	−19	R	Posterior orbital gyrus	GM
51	0.27 (0.23)	−27	−63	65	L	Superior parietal lobule	GM
50	0.27 (0.23)	20	−89	36	R	Cuneus	GM
**Negative**							
5300	0.31 (0.21)	39	−13	67	R	Superior corona radiata and precentral gyrus	WM
4397	0.29 (0.22)	−26	−11	−3	L	Globus pallidus, thalamus, and putamen	GM
3645	0.29 (0.22)	−27	−10	37	L	Superior corona radiata	WM
2106	0.32 (0.22)	18	−3	−7	R	Globus pallidus, thalamus, and putamen	GM
1939	0.32 (0.23)	36	62	2	R	Middle frontal gyrus	GM
1763	0.32 (0.22)	49	−70	−35	R	Cerebellum crus I	GM
696	0.28 (0.22)	2	−5	27	R	Corpus callosum and cingulum	WM
433	0.28 (0.22)	−29	−29	36	L	Superior corona radiata	WM
389	0.28 (0.23)	−49	−16	−1	L	Superior temporal gyrus	WM
349	0.28 (0.23)	−1	−93	10	L	Cuneus	GM
322	0.27 (0.22)	−39	−80	−36	L	Cerebellum crus I	GM
316	0.30 (0.23)	42	57	9	R	Middle frontal gyrus	GM
303	0.26 (0.22)	41	−61	−52	R	Cerebellum lobule VII	GM
230	0.29 (0.24)	−7	56	5	L	Superior frontal gyrus	GM
211	0.25 (0.22)	−24	−56	32	L	Superior parietal lobule	WM
203	0.26 (0.21)	−38	−11	−43	L	Inferior temporal gyrus	GM
150	0.28 (0.22)	−37	50	−4	L	Middle frontal gyrus	WM
124	0.26 (0.23)	−4	−77	−23	L	Cerebellum lobule VII	GM
122	0.25 (0.22)	62	1	17	R	Precentral gyrus	GM
103	0.23 (0.21)	24	−23	8	R	Internal capsule	WM
75	0.24 (0.22)	21	−1	56	R	Superior frontal gyrus	WM
75	0.27 (0.23)	37	−3	30	R	Superior corona radiata	WM
73	0.26 (0.23)	38	49	17	R	Middle frontal gyrus	GM
67	0.25 (0.23)	52	24	10	R	Inferior temporal gyrus	GM
58	0.23 (0.21)	−22	49	0	L	Superior frontal gyrus	WM
54	0.27 (0.24)	28	−19	−25	R	Hippocampal cingulum	WM

Abbreviations: GM, gray matter; L, left; MNI, Montreal Neurological Institute; R, right; WM, white matter.

^a^ Clusters showing significant volumetric differences between the blast-related mild traumatic brain injury and comparison groups are shown. For each cluster, the cluster size, maximum Cohen *d*, MNI coordinates, region, and tissue type are shown.

### Sensitivity Analysis

Analyses including additional covariates were largely consistent (cluster peak Cohen *d* range, −0.22 to −0.40) (eFigure 2 in Supplement 1). Results remained when restricting analyses to individuals with deployment-related mild TBI, comparing blast-related mild TBI with impact mild TBI, when covarying for number of lifetime mild TBIs, and when covarying for time since injury (cluster peak Cohen *d* range, 0.33-0.46). Finally, we created violin plots of cluster volumes across sites at various stages of data adjustment to confirm there were minimal site effects that could bias our results (eFigure 3 in Supplement 1).

### Cognitive Analysis

**Figure 2.**
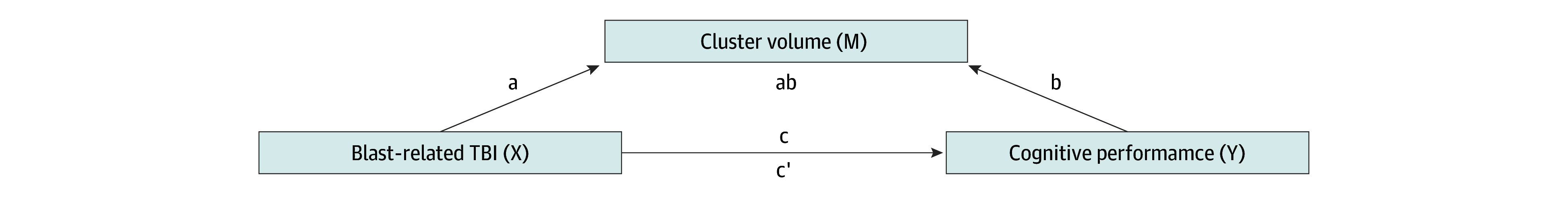
Causal Mediation Analyses Results of causal mediation analyses run in R using the mediation package. For these analyses, the initial variable was blast-related mild traumatic brain injury (TBI) (yes or no), the mediator was volume of either the superior corona radiata or subcortical clusters (derived as described in the Primary Analysis subsection and depicted in Figure 1), and the outcome variables were the Trail-Making Test–B (TMT-B) completion time, the TMT-B and TMT-A completion time, or the Total Digit Span. M mediates the relationship between X and Y.

Across the whole sample, both the Total Recall and Delayed Recall scores of the BVMT-R were positively associated with regional brain volume (mean [SD] score, 8.6 [2.5] for individuals with a history of blast-related mild TBI and 8.6 [2.5] for individuals without a history of blast-related mild TBI [which includes uninjured individuals and those with non–blast-related mild TBI]) (eFigure 4 in Supplement 1). The TMT-B completion time and the TMT-B and TMT-A completion time were negatively associated with volume (mean [SD] TMT-A score, 28.4 [11.4] for individuals with a history of blast-related mild TBI vs 28.0 [10.8] for individuals without a history of blast-related mild TBI; mean [SD] TMT-B score, 66.3 [26.0] for individuals with a history of blast-related mild TBI vs 64.9 [25.7] for individuals without a history of blast-related mild TBI) (shorter completion time = better performance, associated with larger volume; eFigure 5 in Supplement 1). The WAIS-IV Processing Speed Index (mean [SD] score, 19.9 [4.9] for individuals with a history of blast-related mild TBI vs 20.9 [4.9] for individuals without a history of blast-related mild TBI) and the Digit Span performance (mean [SD] score, 27.2 [5.6] for individuals with a history of blast-related mild TBI vs 26.9 [5.1] for individuals without a history of blast-related mild TBI) were positively associated with volume (eFigure 6 in Supplement 1). Within the blast-related mild TBI group, the TMT-B completion time, the TMT-B and TMT-A completion time, and Digit Span yielded clusters that were overlapping with the results from the primary group comparison (eFigure 7 in Supplement 1). The indirect effects for all 6 mediation analyses (subcortical and SCR with TMT-B completion time, TMT-B minus TMT-A completion time, and Digit Span) were significant (mediation coefficient range, 0.49-1.1 for TMT tasks and −0.13 to −0.29 for Digit Span) (Figure 2; Table 3).

**Table 3.** Coefficients for Each Path of Causal Mediation Analyses^a^

Outcome variable	a (Blast volume)	b (Volume outcome)	c (Blast outcome, total effect)	c′ (Blast outcome, total effect)	ab (Effect of blast on outcome through volume)
**Bilateral subcortical cluster volume**					
TMT-B	−0.028^b^	−0.0062^c^	2.3	1.6	0.70^b^
TMT-B and TMT-A		−0.0070^c^	1.2	0.73	0.49^c^
Digit Span		−0.025^c^	0.02	0.15	−0.13^b^
**Bilateral superior corona radiata cluster volume**					
TMT-B	−0.035^d^	−0.0062^c^	2.3	1.2	1.1^b^
TMT-B and TMT-A		−0.0070^c^	1.2	0.53	0.69^c^
Digit Span		−0.034^b^	0/02	0.32	−0.29^d^

^a^ The initial variable was blast-related mild traumatic brain injury (yes or no), the mediator was volume of either the superior corona radiata or subcortical clusters, and the outcome variables were the Trail-Making Test–B (TMT-B) completion time, the TMT-B and TMT-A completion time, or the Total Digit Span. The ab column is the significant indirect effect (see Figure 2).

^b^ *P* < 01.

^c^ *P* < 05.

^d^ *P* < 001.

## Discussion

We found that veterans and active duty service members with a history of blast-related mild TBI have reduced volume in WM and subcortical GM regions compared with those with no history of blast-related mild TBI and that these reduced volumes were associated with decreased processing speed and working memory. Reduced volumes remained after adjusting for covariates including PTSD symptoms, depressive symptoms, and substance use. Further, these findings were unique to blast-related mild TBI, as sensitivity analyses revealed that reduced volumes were not present in veterans and active duty service members with a history of deployment-related impact mild TBI, and this held when controlling for the total number of lifetime TBIs. Overall, these results indicate long-term negative outcomes among veterans with a history of remote blast-related mild TBI (mean [SD] time after injury, 9.8 [5.0] years). Given the high rate of blasts as a mechanism of neurotrauma in modern combat theaters, these results underscore the critical need for continued efforts to mitigate blast-related TBI outcomes, even when TBI severity is mild. Additionally, these highlight the need to identify effective interventions that target blast-related mild TBI pathology and that use currently available and symptom-based targeted clinical interventions, as well as continued prevention strategies, to minimize blast exposure forces in combat and training.

We report smaller volumes in the blast-related mild TBI group in the SCR and in bilateral subcortical clusters that included the globus pallidus, substantia nigra, nucleus accumbens, internal capsule, and cerebellar peduncle. It is well established that WM is particularly vulnerable to disruption after TBI.^62^ The shearing forces of TBI stretch and disrupt axons, causing a chemical and physiological imbalance, or axonopathy with greater focal force.^63,64^ Brain tissue stiffness may be another salient outcome of blast-related mild TBI. Magnetic resonance elastography quantifies these characteristics, and studies have shown that the subcortical GM (in particular the pallidum) and WM (in particular projection tracts such as the corona radiata and internal capsule) have the highest shear stiffness.^65^ Less flexible tissues may be more prone to disruption due to a pressure wave, which may partially underlie the vulnerability of these regions to blast injury.

Several studies have demonstrated cortical thinning in individuals with blast-related mild TBI, primarily in the frontal cortex,^21,22,23^ but volumetric associations are less common.^3,66^ Our work suggests that there are gross alterations in brain volume remotely after blast-related mild TBI, specifically, and contributes to a growing understanding of the unique and adverse long-term effects of blast-related mild TBI on brain function. Mechanisms by which blast-related TBI may effect brain structure include tissue compression and shearing, differential tissue deformation due to varying densities and viscoelastic properties, and expansion of gas-filled spaces (including rupture of the microvasculature).^10^ While we do not have the data to examine cellular mechanisms, any of these could result in tissue loss over time. Many combat and training missions involve high levels of exposure and prolonged exposure to blast waves. Blast exposure is associated not only with a high rate of TBI but also PTSD, personality changes, cognitive deficits, and suicidal behavior.^67^ Our work adds to these earlier findings by showing measurable differences in brain structure associated with differences in cognitive function, unique to blast-related mild TBI.

Mild TBI is associated with changes in cognitive functioning, particularly processing speed and working memory.^68^ However, a neuropathological association between a history of mild TBI and changes in cognitive performance has been less frequently reported. In healthy individuals, the regions implicated in processing speed are lateral frontal and temporal cortices, inferior temporal and parietal cortices, and uncinate fasciculus (connecting frontal and temporal regions).^69^ Working memory performance is similarly supported by prefrontal structures, along with basal ganglia.^70,71,72^ In mild TBI, alterations in WM organization in frontothalamic and frontotemporal tracts are associated with deficits in processing speed and working memory.^73,74,75^ In blast-related mild TBI, reduced cortical thickness in frontal regions is associated with poorer performance on an executive function composite score.^22^ We report that differences in volume partially mediated the association between blast-related mild TBI and processing speed and working memory, providing a potential brain-behavior link. Reductions in processing speed performance involving motor output are often associated with dysfunction in the basal ganglia and frontal cortical-subcortical WM tracts.

### Limitations

Limitations of our study include the retrospective determination of mild TBI–related variables, which is susceptible to self-report error; however, a validated TBI structured interview was used to mitigate this, provide robust standardization, and avoid clinician-level bias in diagnosis. Second, we did not have sufficient data to parse whether subconcussive blast exposures have an additional effect. The LIMBIC-CENC PLS added data collection of the Blast Exposure Threshold Survey^76^ in 2020 to address this limitation, but the current sample size for this analysis was insufficient. Third, the group-based analysis that we conducted will detect only abnormalities that are consistent across a group and thus will miss subject-specific abnormalities. Fourth, as a whole-brain approach, TBM cannot isolate tissue-specific effects. Finally, we were unable to address the effect of adverse childhood experiences, which may affect brain development and could potentially interact with exposures. Adverse childhood experiences were not part of the PLS assessment, but there now is an ongoing effort to collect this information from participants.

### Conclusions

We found that blast-related mild TBI was associated with reduced volume in several central WM and GM structures and that these alterations were further associated with cognitive performance. The results from this cohort study may aid in prognosticating outcomes after injury. By demonstrating the specific cognitive domains that are affected by blast-related mild TBI, our findings may also inform cognitive rehabilitation targets as well as the development of proactive interventions to preserve those functions. Further, these results provide evidence regarding the potential underlying causes of the long-term consequences of blast-related mild TBI and demonstrate that blast-related mild TBI has unique consequences compared with deployment-related mild TBI generally. Our results also indicate that a history of blast-related mild TBI should be continuously considered in regular medical care and any subsequent neuropsychological evaluations. Magnetic resonance elastography studies are needed to look specifically at tissue stiffness and determine whether alterations seen in individuals with blast-related mild TBI are indeed associated with these tissue properties. Changes in military strategy, protective equipment, and/or artillery design are necessary to limit brain damage in military service members. Clinicians treating veterans and active duty service members should not treat a patient’s history of blast-related mild TBI as a static event but should consider the potential chronic and remote adverse effects on neurobehavioral health over the lifespan.^77^

## Notes

Supplement 1. **eAppendix.** Tensor-based morphometry methods **eTable.** Scan parameters for each site **eFigure 1.** Workflow for multi-site template creation (A) and multi-stage registration (B) **eFigure 2.** Overlapping clusters for sensitivity analyses **eFigure 3.** Violin-box plots demonstrating consistency across sites **eFigure 4.** Voxelwise associations with BVMT-R performance across the whole sample **eFigure 5.** Voxelwise associations with Trails performance across the whole sample **eFigure 6.** Voxelwise associations with WAIS-IV performance across the whole sample **eFigure 7.** Voxelwise associations with cognitive performance in the blast mTBI group
eReference.

Supplement 2.
Data Sharing Statement
