# Supplementary material for: Differences in Brain Volume in Military Service Members and Veterans After Blast-Related Mild TBI: A LIMBIC-CENC Study
Source: JAMA Netw Open. 2024 Nov 11;7(11):e2443416. doi: 10.1001/jamanetworkopen.2024.43416 (PMC11555548; doi:10.1001/jamanetworkopen.2024.43416)
Supplement: Supplement 2. — Data Sharing Statement [file jamanetwopen-e2443416-s002.pdf]

Data Sharing Statement

Dennis. Differences in Brain Volume in Military Service Members and Veterans After Blast-Related Mild TBI. *JAMA Netw Open*. Published November 11, 2024. doi:10.1001/jamanetworkopen.2024.43416

Data
Data available: Yes
Data types: Deidentified participant data
How to access data: Access to the data for this study will be possible on request and approval by the LIMBIC-CENC data board (Virginia Commonwealth University, Richmond, Virginia).
When available: With publication

Supporting Documents
Document types: None

Additional Information
Who can access the data: Researchers whose proposed use of the data has been approved
Types of analyses: For a specific purpose
Mechanisms of data availability: After approval of a proposal
